# Downregulation of the CCL2/CCR2 and CXCL10/CXCR3 axes contributes to antitumor effects in a mouse model of malignant glioma

**DOI:** 10.1038/s41598-020-71857-3

**Published:** 2020-09-17

**Authors:** Kenji Shono, Izumi Yamaguchi, Yoshifumi Mizobuchi, Hiroshi Kagusa, Akiko Sumi, Toshitaka Fujihara, Kohei Nakajima, Keiko T. Kitazato, Kazuhito Matsuzaki, Hideyuki Saya, Yasushi Takagi

**Affiliations:** 1 Department of Neurosurgery, Graduate School of Biomedical Sciences, Tokushima University, 3-18-15, Kuramoto-cho, Tokushima, 770-8503 Japan; 2 Division of Gene Regulation, Institute for Advanced Medical Research, School of Medicine, Keio University, Shinjuku-ku, Tokyo, 160-8582 Japan

**Keywords:** Cancer prevention, CNS cancer

## Abstract

Glioblastoma multiforme involves glioma stem cells (GSCs) that are resistant to various therapeutic approaches. Here, we studied the importance of paracrine signaling in the glioma microenvironment by focusing on the celecoxib-mediated role of chemokines C–C motif ligand 2 (CCL2), C-X-C ligand 10 (CXCL10), and their receptors, CCR2 and CXCR3, in GSCs and a GSC-bearing malignant glioma model. C57BL/6 mice were injected with orthotopic GSCs intracranially and divided into groups administered either 10 or 30 mg/kg celecoxib, or saline to examine the antitumor effects associated with chemokine expression. In GSCs*,* we analyzed cell viability and expression of chemokines and their receptors in the presence/absence of celecoxib. In the malignant glioma model, celecoxib exhibited antitumor effects in a dose dependent manner and decreased protein and mRNA levels of *Ccl2* and *CxcL10 and Cxcr3* but not of *Ccr2*. CCL2 and CXCL10 co-localized with Nestin^+^ stem cells, CD16^+^ or CD163^+^ macrophages and Iba-1^+^ microglia. In GSCs, celecoxib inhibited *Ccl2* and *Cxcr3* expression in a nuclear factor-kappa B-dependent manner but not *Ccr2* and *CxcL10*. Moreover, *Ccl2* silencing resulted in decreased GSC viability. These results suggest that celecoxib-mediated regulation of the CCL2/CCR2 and CXCL10/ CXCR3 axes may partially contribute to glioma-specific antitumor effects.

## Introduction

Glioblastoma multiforme (GBM) is the most malignant type of astrocytic glioma and has the highest mortality rate among intracranial tumors. Despite the availability of many therapeutic approaches including surgery, radiotherapy, and chemotherapy, patient prognosis remains poor^1^. GBM involves glioma stem cells (GSCs) that exhibit self-renewal capacity and resistance to chemotherapy and radiotherapy. Although they affect infiltration and proliferation of cancer cells, there are few studies on the pathophysiology of and therapeutic approaches for GSCs.

Recently, increasing attention has been focused on the importance of paracrine-signaling networks within the microenvironment on GSC growth and maintenance^2,3^. Chemokines recruit monocytes to tumor sites, where monocytes are differentiated into tumor-associated macrophages and promote tumor growth, with interactions between chemokines and chemokine receptors proposed as important to cancer initiation and progression^4–8^. C–C motif chemokine ligand 2 (CCL2) and C-X-C motif chemokine ligand 10 (CXCL10) are associated with macrophage infiltration and cell proliferation. CCL2 bound to C–C motif chemokine receptor 2 (CCR2) is involved in promoting cancer angiogenesis, proliferation, and metastasis^4,5^, whereas chemokine CXCL10 binds to C-X-C chemokine receptor 3 (CXCR3) and exhibits dual functions on tumorigenesis depending on the spliced variant: CXCR3-A promotes cell proliferation and CXCR-B exerts growth inhibition^6,7^. A previous study suggested that the expression of chemokine receptor/ligand pairs such as CXCR3/CXCL10 plays an important role in the proliferation of glioma cells^8^. Therefore, we focused on the paracrine-signaling networks involved in the growth microenvironment and maintenance of GSCs related to antitumor effects.

Cyclooxygenase (COX)-2 levels increase in response to various stimuli, and COX-2 increases the malignant potential of human glioma cells^9,10^. A new generation of nonsteroidal anti-inflammatory drugs (celecoxib) selectively inhibit COX-2 activity, which exerts COX-2-dependent and -independent antitumor effects^11^. We recently demonstrated that celecoxib treatment of low-grade glioma cells induces apoptosis and inhibits their proliferation via the Akt/survivin and Akt/inhibitor of differentiation (Id)-3 pathways^12^. Additionally, Kurtova et al.^13^ reported that blocking prostaglandin E2-induced tumor re-population abrogates bladder cancer chemoresistance. Furthermore, Fujita et al.^14^ demonstrated that COX-2 blockade suppresses gliomagenesis by inhibiting myeloid-derived suppressor cells accompanied by chemokine regulation. Moreover, the nuclear factor-kappaB (NF-κB)-dependent cytokine pathway is reportedly associated with mesenchymal glioblastoma^15^.

Based on these findings, we hypothesized that in GSCs and a GSC-bearing malignant glioma model, modulation of the CCL2/CCR2 and CXCL10/CXCR3 axes by celecoxib might contribute to antitumor effects. Therefore, we examined the relationships between celecoxib-specific antitumor effects and regulation of chemokines in a mouse GSC-bearing glioma model that closely resembled human GBM^16^. Furthermore, we studied the mechanisms underlying regulation of the expression of chemokines and their receptors in GSCs. Here, we show that suppression of the CCL2/CCR2 and CXCL10/CXCR3 axes by celecoxib in the tumor and the tumor microenvironment might contribute to antitumor effects.

## Results

### Celecoxib-mediated induction of apoptosis and inhibition of cell proliferation in mouse GSCs

**Figure 1:**
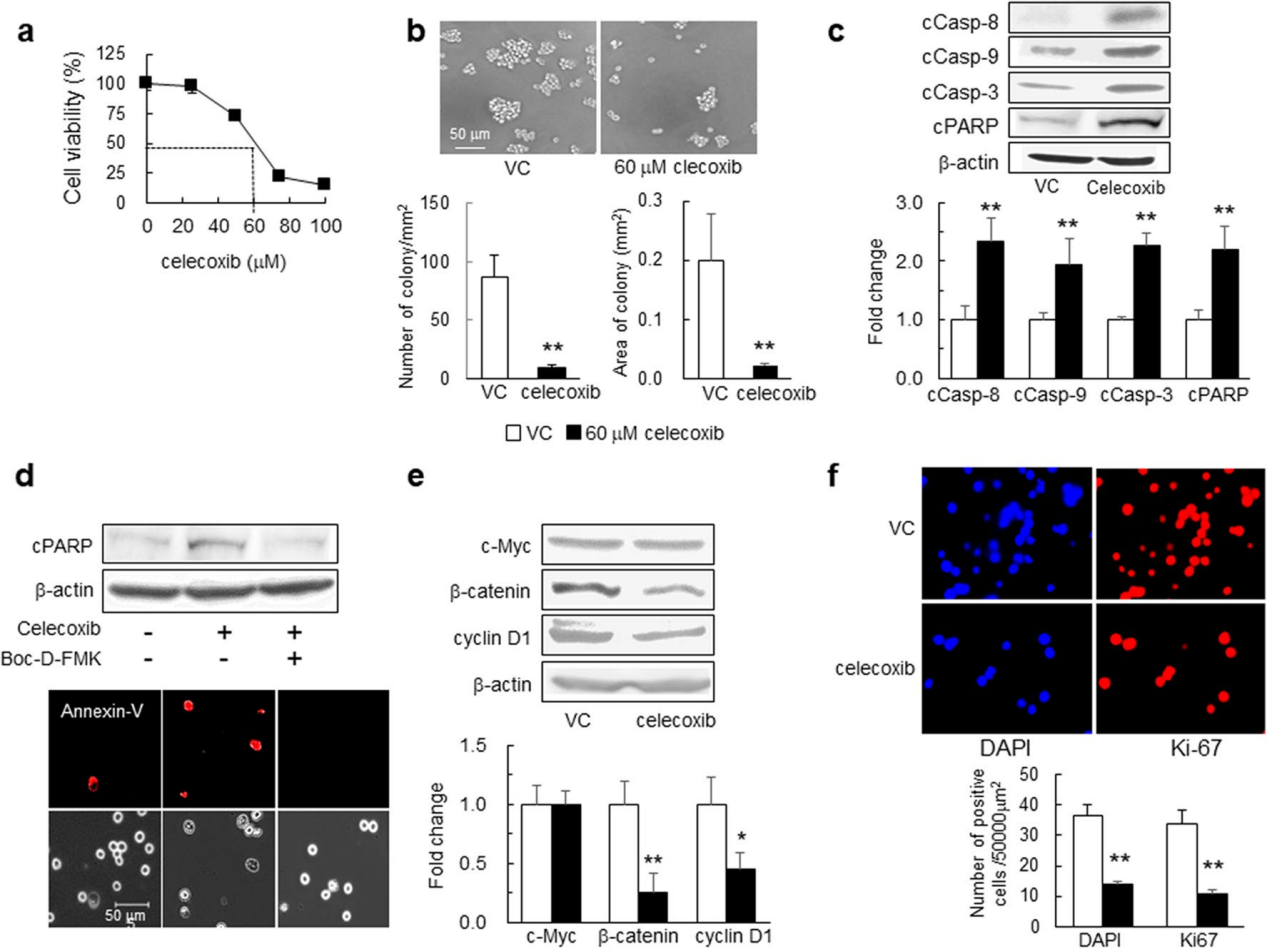
Effects of celecoxib on GSC viability, colony formation, and apoptosis. (**a**) GSC viability evaluated at 24-h post-treatment with celecoxib using water-soluble tetrazolium (WST-8 assay) (*n* = 8). (**b**) Representative images of GSCs 24-h post-treatment with 60 μM celecoxib (50% inhibition concentration; IC_50_) or vehicle control (VC). The number of colony (≥ 2000 μm^2^) and the colony area (mm^2^) per mm^2^ were determined (*n* = 5). (**c**) Representative western blot (WB) images of cleaved caspase (cCasp)-8, -9, and -3 and PARP levels in GSCs treated with 60 μM celecoxib or VC for 6 h (*n* = 4). (**d**) Representative images of WB and Annexin-V (red) staining in GSCs treated with 60 μM celecoxib for 12 h in the presence or absence of 40 μM of the pan-caspase inhibitor Boc-D-FMK (*n* = 3). Scale bar, 50 µm. (**e**) Representative WB images of c-Myc, β-catenin, and cyclin D1 levels in GSCs after treatment with 60 μM celecoxib or VC for 24 h (*n* = 4). (**f**) Ki-67 positive cells (Ki-67^+^, red) detected by immunohistochemistry in GSCs. The number of Ki-67^+^ cells and DAPI^+^ (blue) nuclei was calculated (*n* = 4). Data represent the mean ± SD. *p < 0.05, **p < 0.01 vs. VC by Student’s *t* test.

We first examined the effects of celecoxib on apoptosis-related molecules and cell proliferation. In GSCs exposed to celecoxib for 24 h, cell viability was inhibited in a dose-dependent manner, and the 50% inhibition concentration of celecoxib was 60 μM (Fig. 1a). Additionally, in GSCs treated with 60 μM celecoxib for 24 h, the number and area of colony formation decreased (Fig. 1b). Furthermore, celecoxib increased levels of cleaved caspase-8, -9, and -3 and poly (ADP-ribose) polymerase (PARP) compared with those in the vehicle control (Fig. 1c). The increased PARP cleavage and the number of celecoxib-induced Annexin-V^+^ cells were attenuated after the administration of 40 μM Boc-D-FMK, a pan-caspase inhibitor (Fig. 1d). These results indicated the induction of both intrinsic and extrinsic caspase-dependent apoptosis by celecoxib.

The transcription factor β-catenin and c-Myc play an important role in stem cell renewal and organ regeneration^17^. Additionally, a cell cycle regulator cyclin D1 is linked to cancer development and progression, and acts as a transcription co-regulator^18^. Treatment with 60 μM celecoxib for 24 h reduced levels of β-catenin and cyclin D1 but not of c-Myc (Fig. 1e), and lowered the number of 4′,6-amino-2-phenylindole dihydrochloride (DAPI)^+^ nuclei and Ki-67^+^ cells as a cell-proliferation marker (Fig. 1f). Collectively, these findings suggest that celecoxib might not only induce apoptosis but also inhibit GSC proliferation.

### Chemokine regulation associated with antitumor effects by celecoxib in a mouse malignant glioma model

**Figure 2:**
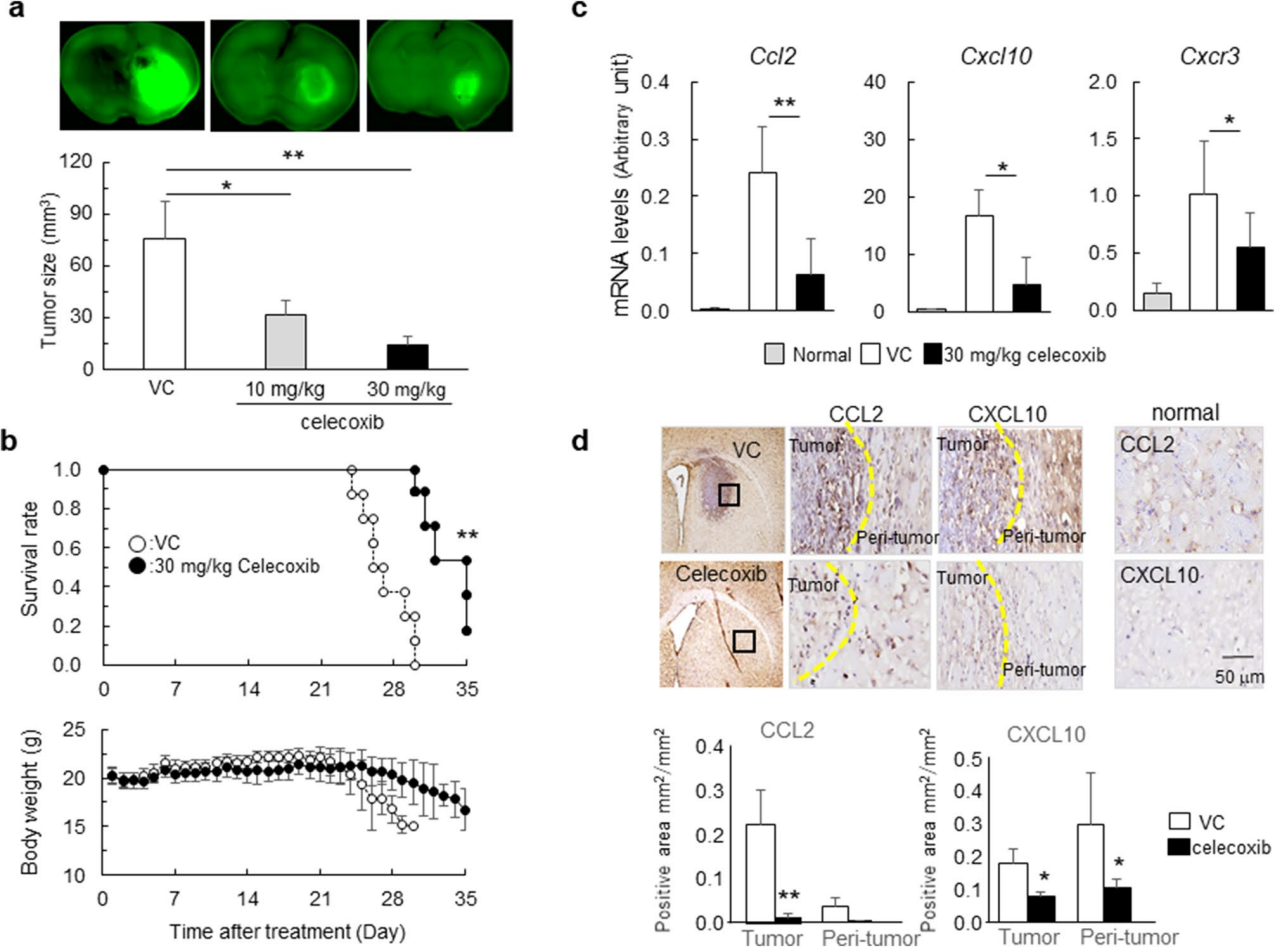
Antitumor effects of celecoxib in the mouse glioma model. (**a**) GFP^+^ tumor areas (green) measured in mice treated with 10 mg/kg/day or 30 mg/kg/day celecoxib or vehicle for 7 days (*n* = 6). *p < 0.05, **p < 0.01 vs. VC by Tukey–Kramer test. (**b**) Survival curves estimated with the Kaplan–Meier method and changes in body weight for 35 days (*n* = 8). **p < 0.01 (log-rank test). (**c**) *Ccl2*, *Cxcl10 and Cxcr*3 mRNA levels determined in mice treated with 30 mg/kg/day celecoxib or VC for 7 days and compared with the normal tissue obtained from age-matched C57BL/6 J mice. *p < 0.05, **p < 0.01 vs. normal by Student’s t-test. (*n* = 6). (**d**) Representative immunohistochemistry stained with DAB in mice treated with 30 mg/kg/day celecoxib or VC for 7 days. Dashed line, tumor border. The positive areas of CCL2 and CXCL10 in tumor and peri-tumor were analyzed by BZ-X710 microscope equipped analyzing system. Scale bar, 50 µm. Each column data indicates mean ± SD (n = 6). *p < 0.05, **p < 0.01 by student’s t-test.

To evaluate the antitumor effects of celecoxib, we administered celecoxib at 10 mg/kg/day or 30 mg/kg/day for 14 days to GSC-bearing glioma models, and found that celecoxib suppressed the tumor volume in a dose-dependent manner (Fig. 2a). Another set of mice treated with 30 mg/kg/day celecoxib or vehicle followed by a 35-day observation period revealed that celecoxib significantly extended the survival period without affecting body weight, whereas all mice in the vehicle control died at day ~ 28 accompanied by a decrease in body weight (Fig. 2b).

To address the mechanisms underlying the antitumor effects of celecoxib, we focused on the tumor microenvironment and examined the levels of chemokines CCL2 and CXCL10 and their receptors, CCR2 and CXCR3, respectively. Treatment with celecoxib notably decreased the mRNA levels of *Ccl2* and *Cxcl10 and Cxcr3* (Fig. 2c) but not of *Ccr2* (data not shown). Immunohistochemically, the expression of CCL2^+^ cells and CXCL10^+^ cells in tumor and peri-tumor were higher than in normal brain tissues, and celecoxib decreased the number of these cells (Fig. 2d).

### Localization of CCL2^+^ cells and CXCL10^+^ cells in a malignant glioma model

**Figure 3:**
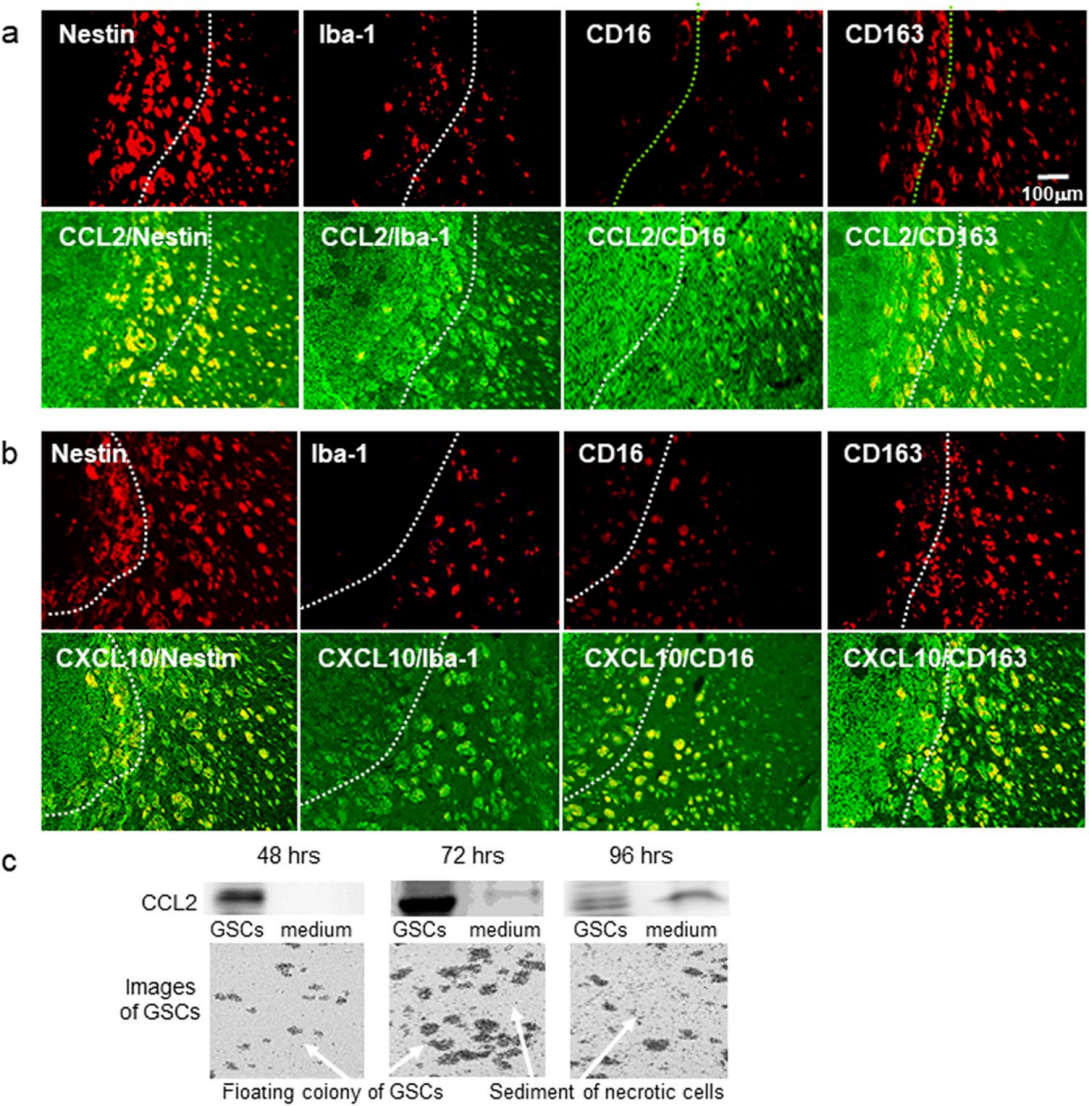
Localization of CCL2^+^ and CXCL10^+^ cells in the mouse glioma model. (**a**) Representative immunohistochemical images of **CCL2**^**+**^ cells (green) localized among Nestin^**+**^, Iba-1^**+**^. CD16^**+**^ or CD163^**+**^ cells (red). (**b**) Representative immunohistochemical images of CXCL10^+^ cells (green) localized with Nestin^**+**^, Iba-1^**+**^. CD16^**+**^ or CD163^**+**^ cells (red). (**c**) Representative image of western blot analysis of CCL2 in GSCs and the culture medium. The experiment was repeated 3 times. The cell image was taken by BZ-X710 microscope. Each culture medium was centrifuged using Microcon Centrifugal Filters for protein concentration.

Annovazzi et al.^19^ reported that glioma-associated microglia macrophages (GAMs) assimilated to peripheral macrophages are polarized and have two functional profiles: pro-inflammatory M1-type- and immunosuppressive, anti-inflammatory M2-type macrophages. The phenotype of GAMs overlaps only partially with M1 and M2 phenotypes. To identify GAMs, 4 antibodies are currently used: Iba1, CD16, CD68, and CD163^20^. To analyze which cells co-localized with CCL2^+^ or CXL10^+^ cells in the glioma model, we used the stem cell marker Nestin, a microglia marker Iba-1, and the M1- and M2-type monocyte macrophage markers CD16 and CD163, respectively. As shown in Fig. 3a,b, we found that in the tumor and the tumor microenvironment, CCL2 and CXCL10 localized in Nestin^+^, Iba-1^+^, CD16^+^ and CD163^+^ cells.

Since CCL2 is secreted by necrotic and endothelial cells^21^, we analyzed CCL2 expression during culture for 48–96 h. (Fig. 3C). GSCs proliferated in a time-dependent manner. In the presence of dead cells, CCL2 expression increased in the culture medium as well as in GSCs. The expression of CCL2 in the culture medium increased in the presence of dead cells may suggest the secretion of CCL2 from necrotic GSCs.

### Downregulation of CCL2 and CXCR3 via the NF-κB pathway by celecoxib inhibits GSC viability

**Figure 4:**
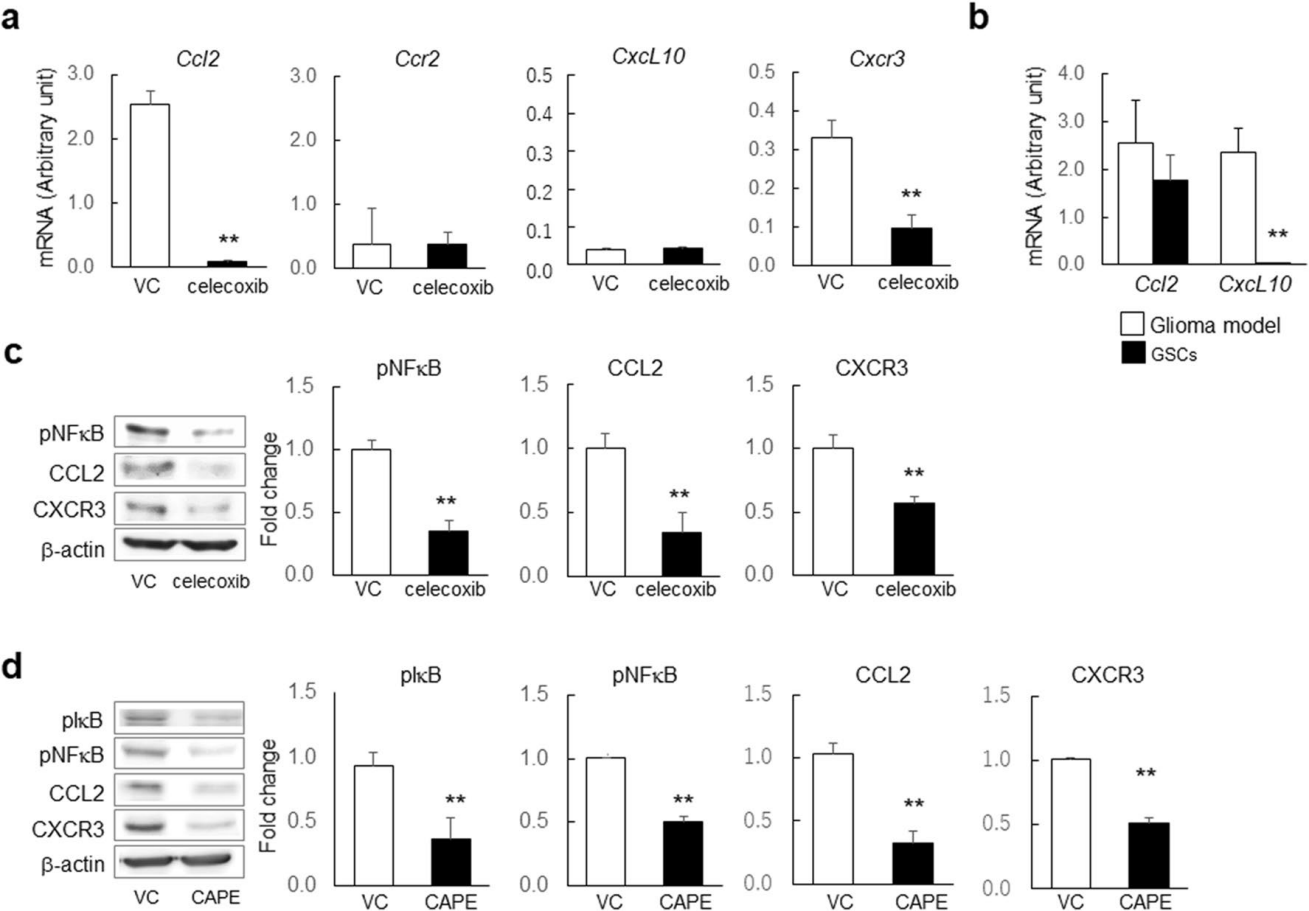
Effect of celecoxib treatment on *Ccl2, Cxcl10, Ccr2*, and *Cxcr3* expression in mouse GSCs. (**a**) mRNA levels of *Ccl2*, *Cxcl10*, *Ccr2,* and *Cxcr3* determined in mouse GSCs treated with 60 µM celecoxib or vehicle control (VC) for 24 h (n = 6). (**b**) Comparison of mRNA levels of *Ccl2* and *Cxcl10* in the mouse glioma model and GSCs (n = 6). (**c**) Representative images of western blot analysis and the expression levels of phosphorylated NF-κB (pNF-κB), CCL2 and CXCR3 in mouse GSCs treated with VC or celecoxib (*n* = 4). (**d**) Representative images of western blot analysis and the expression levels of phosphorylated IκBα (pIκBα), pNF-κB, CCL2, and CXCR3 in mouse GSCs treated with VC, and CAPE (*n* = 4). Data represent the mean ± SD. **p < 0.01 vs. VC by Student’s *t* test.

We addressed the regulation of chemokines (CCL2 and CXCL10) and their receptors CCR2 and CXCR3) in GSCs treated with 60 µM celecoxib for 24 h in GSCs. In GSCs, celecoxib revealed significant decrease in mRNA levels of *Ccl2* and *Cxcr3* but not of *Ccr2* and *Cxcl10* (Fig. 4a), which was consistent with the high mRNA levels of *Ccl2* in the glioma model and GSCs but none of *Cxcl10* in GSCs (Fig. 4b). The protein expression of pNFkB, Ccl2, and Cxcr3 was decreased (Fig. 4c) without affecting CCR2 and CXCL10 (data not shown). Then, using caffeic acid phenethyl ester (CAPE), which is a potent NF-κB inhibitor that suppresses the translocation of NF-κB p65 from the cytoplasm to the nucleus, we confirmed that the levels of pIκB, pNF-kB, CCL2, and CXCR3 were significantly decreased in GSCs (Fig. 4d). The proteasomal degradation of IkBa is a key step in the regulation of the NF-kB pathway and liberates the NF-kB proteins from IkBa, leading to their nuclear translocation and increasing their interaction with NF-kB DNA-binding properties to result in the activation of gene expression^22^. These results suggested NF-kB-dependent transcriptional regulation of CCL2 and CXCR3 levels by celecoxib (Fig. 4d).

**Figure 5:**
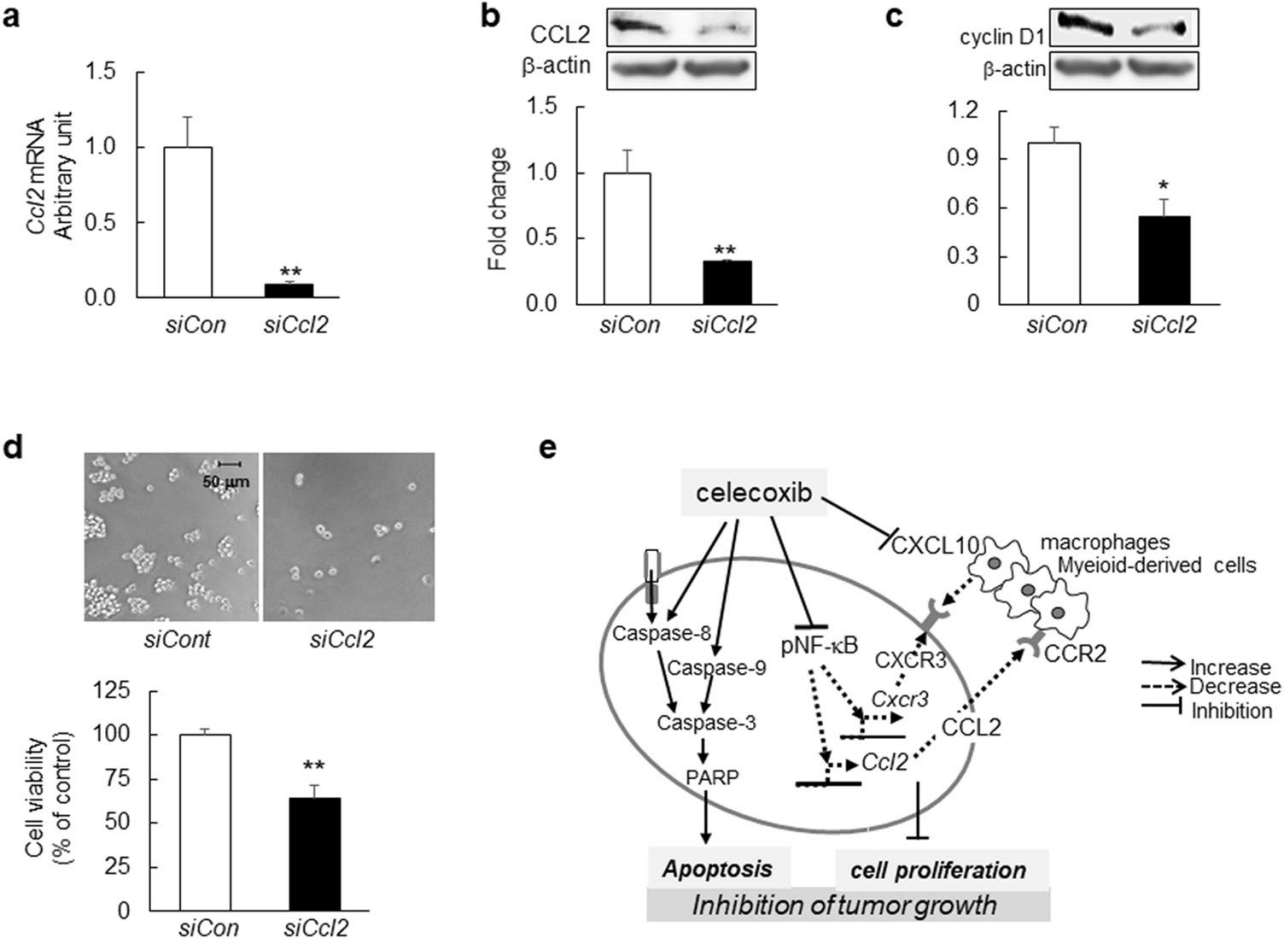
GSC viability following *Ccl2* silencing. (**a**) *Ccl2* mRNA levels in GSCs transfected with 10 nM *Ccl2* siRNA (siCcl2) or negative control siRNA (siCon) for 24 h (*n* = 6). (**b**) Representative western blot results for CCL2 (*n* = 4). (**c**) Representative western blot results for cyclin D1 from GSCs at 48-h post-transfection with siCcl2 or siCon (*n* = 4). (**d**) Representative images of GSCs and cell viability evaluated by WST-8 assay at 48-h post-transfection with siCcl2 or siCon (*n* = 4). Scale bar, 50 µm. (**e**) Schematic illustration of the antitumor effects of celecoxib in the mouse glioma model. Data represent the mean ± SD. *p < 0.05, **p < 0.01 vs. siCon by Student’s *t* test.

Finally, we assessed the functional relevance of CCL2 in GSCs. *Ccl2* silencing by small-interfering (si)RNA decreased *CCL2* mRNA and protein levels (Fig. 5a,b) 24 h after transfection. Additionally, 48 h after transfection, cyclin D1 levels decreased (Fig. 5c) accompanied by decreased GSC viability (Fig. 5d), suggesting that reduced CCL2 level might be partly associated with GSC growth inhibition.

## Discussion

This is the first study showing that the suppression of the CCL2/CCR2 and CXCL10/CXCR3 axes by celecoxib may attribute to antitumor effects in a mouse malignant glioma model, in addition to intrinsic and extrinsic apoptotic induction by celecoxib in GSCs (Fig. 5e). We newly found that in the mouse glioma model, celecoxib decreased mRNA levels of *Ccl2, CxcL10 and Cxcr3 but not of Ccr2* and protein expression of CCL2 and CXCL10 in the tumor and peri-tumor. Moreover, we demonstrated that in GSCs, celecoxib treatment decreased protein and mRNA level of *Ccl2* and *Cxcr3* levels without affecting *Ccr2* and *CxcL10*, which were associated with the inhibition of NF-κB signaling, and that *Ccl2* silencing by siRNA inhibited cell proliferation. These results showed that celecoxib-induced the modulation of chemokine and chemokine-receptor levels in GSCs and the tumor microenvironment may be at least partly attributable to its antitumor effects in a mouse malignant glioma model. GBM comprises a heterogeneous cell population that includes tumor and immune cells, with interactions among these different cell types and chemokines/cytokines promoting tumor development and progression^20^. CCL2 is a major chemokine involved in attracting bone-marrow-derived mesenchymal cells to the tumor microenvironment, and CCL2 loss decreases accumulation of these cells^21^. In the present study, we identified CCL2 in GSCs, the mouse glioma model, and in the culture medium in the presence of a large number of dead cells. Moreover, CCL2 and CXCL10 was present in the tumor and the tumor microenvironment and co-localized with almost all Nestin^+^, Iba-1^+^, CD163^+^ or CD16^+^ cells, suggesting the same distribution and localization. However, in GSCs*,* the high protein and mRNA levels of *Ccl2* and *Cxcr3* were decreased by celecoxib, whereas the low protein and mRNA level of *Ccr2*, *Cxcl10* were not affected by celecoxib in our in vitro study. On the other hand, in the mouse glioma model, the high protein and mRNA level of *Ccl2*, *Cxcl10* and *Cxcr3* were decreased by celecoxib. These findings suggest that CCL2 and CXCL10 and their receptors may be generated from different cell types and have distinct crucial role (Fig. 5e) in tumor growth. The downregulation of these chemokines and receptors by celecoxib might contribute to antitumor effects in both tumor and peri-tumor areas.

Jung et al.^23^ reported that CCL2/MCP1 is secreted by necrotic glioma cells and endothelial cells in glioblastoma. We found high expression levels of CCL2 in GSCs and GSCs-derived glioma model. To demonstrate the secretion of CCL2 by glioma cells, we extended the culture period as shown in Fig. 3c. In the presence of dead cells, the GSCs proliferated in a time-dependent manner, and we observed the increase in CCL2 expression in the culture medium as well as in GSCs. Takeshima et al.^24^ reported that the degree of macrophage infiltration found among the glioma cells was grossly correlated with the level of CCL2/MCP1 expression, suggesting that CCL2/MCP1 produced by the glioma cells may mediate macrophage infiltration into glioma tissues. These findings support our results and a potential therapeutic approach to reduce glioma growth through promoting M1-polarized macrophages and inhibiting M2-phenotypic macrophages^25,26^.

Continuous treatment with CCL2-neutralizing antibodies inhibits tumor progression in hepatocellular carcinoma^27^. CCL2 binds the cognate receptor CCR2, and together this signaling pair has been shown to have multiple pro-tumorigenic roles^28^. Because *Ccl2* mRNA levels are not reduced by treatment with a CCL2 antibody, the recovery of CCL2 level after cessation of CCL2 antibody treatment is suggested. In the present study, NF-κB signaling mediated by celecoxib decreased mRNA and protein levels of CCL2, suggesting greater therapeutic potential than therapy involving the CCL2 antibody. However, celecoxib treatment did not alter CCR2 in the glioma model, we could not clarify the role of CCR2.

Although CXCL10 mediates the chemotaxis of tumor-promoting cells^29^ and exerts dual effects depending on the splicing variant of its receptors^7^, CXCL10 induces DNA synthesis and might promote proliferation of anaplastic astrocytoma (grade III) and GBM^8^. Although CXCL10-specific effects against glioma associated with celecoxib treatment might differ depending on the circumstances^7^, we observed that increased levels of CXCL10^+^ cells in the tumor and peri-tumor, and that CXCL10 levels decreased following treatment with celecoxib in our glioma model. These findings in the glioma model are similar to a previous study showing that CXCL10 is produced in bone-marrow-derived mesenchymal stem cells induced by hypoxia^30^. Therefore, we were unable to address its regulatory mechanisms in GSCs. In contrast, we observed reduced CXCR3 levels associated with attenuated NFκB signaling in GSCs. Because increased CXCR3 levels are indicative of poor survival rates, and targeted silencing of *Cxcr3* inhibits the proliferation of hepatocellular carcinoma cells^31^, these findings support the present results showing that celecoxib-specific inhibition of growth and/or proliferation might partially contribute to suppression of the CXCL10/CXCR3 axis in GSCs.

The tumorigenic potential of increased COX-2 levels is linked to apoptosis resistance in some cell types, and celecoxib-induced apoptosis involves the extrinsic/death receptor- and/or intrinsic/mitochondria-mediated pathways^32^. In agreement with these findings, we found that celecoxib treatment increased caspase-8, -9, and -3 levels, as well as those of PARP levels in GSCs, indicating that apoptosis occurred via both pathways. Moreover, β-catenin and cyclin D1 levels were decreased, and Ki-67 level were reduced by celecoxib treatment, which agreed with a study of human glioma cell lines treated with celecoxib^33^. Therefore, the antitumorigenic effects of celecoxib might include not only induced apoptosis but also suppressed cell proliferation (Fig. 5e).

We initiated celecoxib treatment on day 1 following intracranial GSC injection, which is akin to celecoxib administration after surgical resection of human gliomas. Our results suggest the possibility that glioma patients having undergone tumor resection might benefit from celecoxib treatment to reduce the risk of GSC-driven tumor growth.

There are some limitations to this study. First, our GSCs were established from mouse neural stem cells, and this animal model exhibited pathological similarities to human GBM.^16^ with a more accurate reflection of its features and malignant behavior. However, the glioma in the mouse model might differ from that of human gliomas, which are heterogeneous, unlike the homogeneous GSCs derived from oncogene knock-in mice. Second, we analyzed the effects of celecoxib treatment using only one cell line derived from mice and one animal model. Further studies using GSCs derived from humans are required. Moreover, a number of distinct molecular pathways might play a role in the antitumor effects of celecoxib in a COX-2-dependent and/or -independent manner^11^. Because previous studies suggested alternative targets implicated in the tumor-inhibitory effects of celecoxib, we cannot rule out other targets associated with its antitumor effects.

In conclusion, our findings suggest that downregulation of the CCL2/CCR2 and CXCL10/CXCR3 axes via inhibition of the NF-κB pathway in the tumor and the tumor microenvironment might have contributed to the antitumor effects of celecoxib observed in the mouse glioma model. These results warrant further investigation to clarify the therapeutic benefits of celecoxib in humans.

## Materials and methods

All experiments and protocols were approved by the ethics committee of the Graduate School of Biomedical Sciences, Tokushima University (Tokushima, Japan).

### Cell line

GSCs were established by Saya et al.^16^ and comprised H-Ras^V12^ overexpressed in *Ink4a/Arf*-null neural stem cells and the progenies of GSCs were maintained as a sphere culture in Dulbecco’s modified Eagle medium F-12 (Sigma-Aldrich, St. Louis, MO, USA) supplemented with 20 ng/mL recombinant human epidermal growth factor (PeproTech, Rocky Hill, NJ, USA), 20 ng/mL recombinant human basic fibroblast growth factor (PeproTech), B-27 supplement without vitamin A (Life Technologies, Carlsbad, CA, USA), 200 ng/mL heparin sulfate, 100 U/mL penicillin, and 100 μg/mL streptomycin (Nacalai Tesque, Kyoto, Japan) at 37 °C in an atmosphere of 5% CO_2_/95% humidified air.

### Cell viability and colony formation assays

GSCs were seeded in 96-well culture plates (1 × 10^4^ cells/well). Celecoxib was purchased from Sigma-Aldrich (Tokyo, Japan) which was dissolved in dimethyl sulfoxide (DMSO) and diluted to 0.1% with culture medium for use as the vehicle control. The number of viable cells was determined using a Cell Counting Kit-8 (Dojindo Laboratories, Kumamoto, Japan) and measured on an Infinite F200 Pro microplate reader (Tecan, Mannedorf, Switzerland) at 450 nm. Cell viability following celecoxib treatment was calculated as the percentage of the vehicle control. For the colony-formation assay, GSCs were seeded in 96-well plates (5 × 10^3^ cells/well) and treated with celecoxib or vehicle for 24 h. The number of colonies with size > 2000 μm^2^ and the area of colony were analyzed using a BZ-X710 microscope (Keyence, Osaka, Japan) equipped image analysis system.

### Animal experiments

Experiments were performed according to the National Institutes of Health Guide for the Care and Use of Laboratory Animals. The GSC-bearing glioma model, which resemble human malignant glioma, was previously established by Saya et al.^16^ and used according to their protocol.

Male C57BL/6 J mice (6-weeks old) were obtained from CLEA Japan (Tokyo, Japan). Animals were anesthetized and placed in a stereotaxic apparatus, and a small hole was bored into the skull of each mouse (2.0-mm lateral to bregma) using a dental drill. Viable cells (1 × 10^3^) resuspended in 2 μl of Hank’s balanced salt solution were injected into the right hemisphere 3-mm below the brain surface using a 10-μL Hamilton syringe with an unbeveled 30-gauge needle. The injection was performed over 5 min, and the needle was left in the brain for an additional 5 min before it was gradually withdrawn and the hole sealed. Celecoxib was dissolved in DMSO and diluted four-fold with sterile water immediately before injection. Celecoxib doses were established based on our previous study^12^. Treatment with 10 mg/kg or 30 mg/kg celecoxib or vehicle (control) by intraperitoneal injection was initiated on the first day of GSC injection. To determine tumor size, fixed brain tissue was sliced on a brain-slicer matrix at 1.0-mm intervals. Based on the tumor area, represented by the green fluorescent protein (GFP)^+^ area in the slice, the tumor volume was calculated using a microscope equipped with an image analysis system (BZ-X710; Keyence).

### Western blot analysis

Protein samples were separated by sodium dodecyl sulfate–polyacrylamide gel electrophoresis and transferred to a polyvinylidene difluoride membrane (Bio-Rad, Hercules, CA, USA) that was incubated in blocking solution. Membranes were incubated with primary antibodies (Supplementary Table 1), followed by incubation with horseradish peroxidase-conjugated secondary antibody. Immunoreactivity was detected using enhanced chemiluminescence prime western blot detection reagent and visualized with an Image Quant LAS4000 mini (GE Healthcare, Little Chalfont, UK). Band intensities were quantified using ImageJ software (v.1.49; National Institutes of Health, Bethesda, MD, USA) and normalized to β-actin. To detect extracellular CCL2 in the culture medium, we collected the medium containing proliferated GSCs at 48, 72, and 96 h after cell culture. The medium was fractionated according to molecular weight using Microcon centrifugal filters (Merk, Tokyo, Japan) for protein concentration. An inhibitor of NF-κB (Caffeic acid phenethyl ester; CAPE; R&D System, Minneapolis, MN, USA) was dissolved in DMSO.

### Quantitative real-time polymerase chain reaction (qRT-PCR)

Total RNA was purified using a MagNA Pure compact RNA isolation kit (Roche Diagnostics, Burgess Hill, UK) according to manufacturer instructions. cDNA was synthesized using Transcriptor Universal cDNA master mix (Roche Diagnostics), and qRT-PCR was performed using FastStart DNA Master^PLUS^ SYBR Green I (Roche Diagnostics) on a Light Cycler 2.0 instrument (Roche Diagnostics) under conditions recommended by the manufacturer. The forward and reverse primer sequences were as follows: mouse *Ccl2*, 5′-AGCAAGATGATCCCAATGAGT-3′ and 5′-GAGCTTGGTGACAAAAACTACAG-3′; mouse *Cxcl10*, 5′-CCCACGTGTTGAGATCATTG-3′ and 5′-CAGTTAAGGAGCCCTTTTAGACC-3′; mouse *Ccr2*, 5′-CAAAATGGATGCCTTAGCACTG-3′ and 5′-CCAGGTTTTCATGTTTGGCTATTC-3′; mouse *Cxcr3*, 5′-CCAAGCCATGTACCTTGAGGTTAG-3′ and 5′-GCTCTCGTTTTCCCCATAATCGTAG-3′; mouse glyceraldehyde-3-phosphate dehydrogenase (*Gapdh*), 5′-CAGAACATCATCCCTGCATC-3′ and 5′-CTGCTTCACCACCTTCTTGA-3′. mRNA levels were normalized to that of *Gapdh*.

### Small-interfering RNA (siRNA) transfection

Transfections were conducted using Lipofectamine RNAiMAX transfection reagent (Life Technologies) according to manufacturer instructions. Briefly, siRNA and transfection reagent were diluted in OPTI-MEM medium (Life Technologies), and transfection reagent/siRNA complexes were allowed to form for 20 min. The complexes were added to each well, and GSCs were seeded into 6-well culture plates (2 × 10^5^ cells/well) or 96-well culture plates (4 × 10^3^ cells/well) and incubated for 24 h or 48 h. The target sequence for mouse *Ccl2* siRNA (QIAGEN, Valencia, CA, USA) was as follows: 5′-ACCCGTAAATCTGAAGCTAAT-3′. Non-targeting siRNA was used as a negative control.

### Annexin-V assay

Apoptotic cells were labeled with the Annexin-V-PE assay kit (BioVision, Milpitas, CA, USA) according to manufacturer instructions. Briefly, cells were treated with 0.2% DMSO or 60 μM celecoxib and the pan-caspase inhibitor boc-aspartyl-(OMe)-fluoromethyl-ketone (Boc-D-FMK) (40 μM; Abcam, Cambridge, UK) to confirm caspase-dependent apoptosis.

### Immunocytochemistry and immunohistochemistry

GSCs were collected and smeared using Smear Gel (GenoStaff, Tokyo, Japan) according to manufacturer instructions and then fixed with 4% paraformaldehyde (PFA) in phosphate-buffered saline (PBS). After permeabilization with 0.2% Triton X-100 in PBS, cells were labeled with antibody of Ki-67 as a cell proliferation marker (Abcam), the antigen was detected with Alexa Fluor 594-conjugated donkey antirabbit IgG (Life Technologies), and nuclei were counterstained with 4′,6-diamino-2-phenylindole dihydrochloride (DAPI) solution (Dojindo Laboratories).

Tissue samples were fixed with 4% PFA in PBS, and 10-µm-thick frozen sections were mounted on glass slides. For immunoperoxidase staining, sections were blocked and then incubated overnight with primary antibodies. Antibodies were visualized using a VECTASTAIN Elite ABC kit (Vector Laboratories, Burlingame, CA, USA), H_2_O_2_/diaminobenzidine (Merck Millipore, Billerica, MA, USA), and Mayer’s hematoxylin solution according to manufacturer instructions. For double-immunofluorescence staining, sections were immunoreacted with an antibody for nestin-, IBA-1-, CD163 and CD16 positive cells followed by incubation with anti-CCL2 and anti-CXCL10 antibodies. To visualize each marker, sections were incubated with green fluorescence and chemokines with red fluorescence, and sections were incubated with an Alexa 488- or Alexa 594-conjugated secondary antibody (Life Technologies), respectively. Nuclei were counterstained with DAPI solution (Dojindo Laboratories). The detailed information for each antibody is provided in Supplementary Table 2. All images were obtained using BZ-X710 microscope (Keyence).

### Statistical analysis

Data are presented as the mean ± standard deviation (SD) and were analyzed with a Student’s t test for two-group comparisons and analysis of variance, followed by the Tukey–Kramer test for multiple comparisons. A survival curve was estimated by the Kaplan–Meier method, and statistical significance was tested with the log-rank test. Differences between groups were considered statistically significant at p < 0.05.

## Supplementary information

Supplementary information.
