# Supplementary material for: Downregulation of the CCL2/CCR2 and CXCL10/CXCR3 axes contributes to antitumor effects in a mouse model of malignant glioma
Source: Sci Rep. 2020 Sep 17;10:15286. doi: 10.1038/s41598-020-71857-3 (PMC7499211; doi:10.1038/s41598-020-71857-3)
Supplement: Supplementary file 1 — Supplementary information. [file 41598_2020_71857_MOESM1_ESM.pdf]

Downregulation of the CCL2/CCR2 and CXCL10/CXCR3 axes contributes to antitumor effects in a mouse model of malignant glioma

Kenji Shono, Izumi Yamaguchi, Yoshifumi Mizobuchi, Hiroshi Kagusa, Akiko Sumi, Toshitaka Fujihara, Kohei Nakajima, Keiko T Kitazato, Kazuhito Matsuzaki, Hideyuki Saya, Yasushi Takagi

Supplementary Table 1. Antibodies used for western blot.

Antibody	Manufacturer	Dilution	Species	Blocking solution
Cleaved caspase - 3 (Asp175)	Cell Signaling Technology, MA, USA	1:1000	Rabbit	5% skim milk
Cleaved caspase - 8 (Asp387)	Cell Signaling Technology, MA, USA	1:1000	Rabbit	5% skim milk
Cleaved caspase - 9	Cell Signaling Technology, MA, USA	1:1000	Rabbit	5% skim milk
Cleaved PARP (Asp214)	Cell Signaling Technology, MA, USA	1:1000	Rabbit	5% skim milk
β-catenin	BD Biosciences, NJ, USA	1:2000	Mouse	5% skim milk
ϵ Myc	Abcam, Cambridge, UK	1:2000	Rabbit	5% skim milk
Cyclin D1	Santa Cruz Biotechnology, CA, USA	1:500	Rabbit	5% skim milk
CCL2	Abcam, Cambridge, UK	1:1000	Rabbit	5% skim milk
CXCL10	Abcam, Cambridge, UK	1:1000	Mouse	5% skim milk
Phospho-NF-κB p65 (Ser536)	Cell Signaling Technology, MA, USA	1:1000	Rabbit	3% BSA
Phospho-IκBα p65 (Ser32)	Cell Signaling Technology, MA, USA	1:1000	Rabbit	3% BSA
β–actin	Sigma Aldrich MO, USA	1:5000	Mouse	5% skim milk

Supplementary Table 2. Antibodies used for immunofluorescence staining.

Antibody	Manufacturer	Dilution	Species
CCL2	Abcam, Cambridge, UK	1:100	Rabbit
CXCL10	Abcam, Cambridge, UK	1:100	Mouse
Nestin	Abcam, Cambridge, UK	1:100	Mouse
Nestin	Proteintech, USA	1:100	Rabbit
CD163	Abcam, Cambridge, UK	1:100	Mouse
CD163	Abcam, Cambridge, UK	1:100	Rabbit
CD16	Abcam, Cambridge, UK	1:100	Mouse
CD16	Abcam, Cambridge, UK	1:100	Rabbit
Iba-1	Abcam, Cambridge, UK	1:100	Mouse
Iba-1	Abcam, Cambridge, UK	1:100	Rabbit
